# Augmenter of liver regeneration

**DOI:** 10.1186/1755-1536-5-10

**Published:** 2012-07-09

**Authors:** Chandrashekhar R Gandhi

**Affiliations:** 1 VA Pittsburgh Healthcare System, Pittsburgh, PA, USA; 2 Thomas E. Starzl Transplantation Institute, Departments of Surgery and Pathology, University of Pittsburgh, E-1540 BST, 200 Lothrop street, Pittsburgh, PA, 15213, USA

**Keywords:** ALR, GFER, Growth, Liver, Regeneration, Hepatocytes, Survival

## Abstract

‘Augmenter of liver regeneration’ (ALR) (also known as hepatic stimulatory substance or hepatopoietin) was originally found to promote growth of hepatocytes in the regenerating or injured liver. ALR is expressed ubiquitously in all organs, and exclusively in hepatocytes in the liver. ALR, a survival factor for hepatocytes, exhibits significant homology with ERV1 (essential for respiration and viability) protein that is essential for the survival of the yeast, *Saccharomyces cerevisiae*. ALR comprises 198 to 205 amino acids (approximately 22 kDa), but is post-translationally modified to three high molecular weight species (approximately 38 to 42 kDa) found in hepatocytes. ALR is present in mitochondria, cytosol, endoplasmic reticulum, and nucleus. Mitochondrial ALR may be involved in oxidative phosphorylation, but also functions as sulfhydryl oxidase and cytochrome c reductase, and causes Fe/S maturation of proteins. ALR, secreted by hepatocytes, stimulates synthesis of TNF-α, IL-6, and nitric oxide in Kupffer cells via a G-protein coupled receptor. While the 22 kDa rat recombinant ALR does not stimulate DNA synthesis in hepatocytes, the short form (15 kDa) of human recombinant ALR was reported to be equipotent as or even stronger than TGF-α or HGF as a mitogen for hepatocytes. Altered serum ALR levels in certain pathological conditions suggest that it may be a diagnostic marker for liver injury/disease. Although ALR appears to have multiple functions, the knowledge of its role in various organs, including the liver, is extremely inadequate, and it is not known whether different ALR species have distinct functions. Future research should provide better understanding of the expression and functions of this enigmatic molecule.

## Review

### Introduction

Following the discovery that the liver possesses a remarkable ability to regenerate, pursuit to identify factors that are involved in this phenomenon led to the discovery of many mitogens, co-mitogens, and inhibitors. Among them was a protein initially named hepatic stimulatory substance or hepatopoietin and now commonly known as ‘Augmenter of liver regeneration’ (ALR). ALR was subsequently purified, and cloned in rat, mouse, and human. Both native and cloned ALR augment liver regeneration following partial hepatectomy and prevent pathology of portacaval shunt surgery in animal models. ALR is produced and secreted exclusively by hepatocytes in the liver and stimulates synthesis of TNF-α, IL-6, and nitric oxide in Kupffer cells via a G-protein coupled receptor. ALR may also exert actions on hepatocytes in an autocrine manner. Interestingly, intracellular ALR was found to be a survival factor as its depletion causes rapid mitochondrial dysfunction and apoptotic/necrotic death of hepatocytes. In this regard, ALR exhibits significant homology with ERV1 protein (essential for respiration and viability found in the yeast, *Saccharomyces cerevisiae*). Thus it is not a surprise that ALR is expressed ubiquitously in all organs, and may have tissue-specific functions. Furthermore, post-translational modification of the 22 kDa native ALR protein to three high molecular weight species (38 to 42 kDa), and presence of ALR in mitochondria, cytosol, endoplasmic reticulum, and nucleus indicate that ALR may play an important role in various physiological functions in a cell. Current evidence indicates that ALR may be involved in mitochondrial oxidative phosphorylation, reduction of cytochrome c, and in regulation of the activities of certain proteins by its sulfhydryl oxidase activity as well as by inducing Fe/S maturation of proteins. Thus, although ALR appears to have multiple functions, the knowledge of its roles in various organs, even in the liver, is very inadequate. In this article, the history and the current status of ALR research is reviewed. The intent of this summary is to stimulate further research to unravel the many facets of the important functions of this enigmatic protein in physiology and pathology.

### History of augmenter of liver regeneration

Liver regeneration is a fascinating phenomenon that occurs following hepatocellular death induced by toxins, viral infection, physical injury due to trauma or elective resection for focal disease, and following transplantation. The ability of the liver to regenerate is known since the myth of Prometheus whose liver, fed to an eagle persistently as a punishment for stealing fire from Zeus and giving it to mortals, regenerated in perpetuity. The scientific proof that the liver indeed possesses regenerative ability was provided by Higgins and Anderson [1] when they demonstrated restoration of the liver mass following partial hepatectomy in rats. Subsequent research led to the discovery of several endogenously and exogenously produced growth promoters (for example, HGF, TGF-α, epidermal growth factor [EGF], platelet-derived growth factor [PDGF]), co-mitogens/priming agents (for example, insulin, insulin-like growth factor [IGF], IL-6, TNF-α), growth inhibitors (such as TGF-β, activins), intrinsic molecular mechanisms, and signaling pathways that participate in hepatocyte proliferation following partial resection or death of hepatocytes due to physical, chemical or biological injury For review, see [2-5].

Early attempts to identify hepatocyte mitogens led to the discovery that factors present in the soluble fractions of regenerating liver or in the serum of partially hepatectomized rats stimulated liver regeneration *in vivo* and DNA synthesis in hepatocytes *in vitro*[6-9]. In 1975, Labreque and Pesch [10] identified a protein in weanling and regenerating rat livers that stimulated liver regeneration, and named it ‘hepatocyte stimulatory substance (HSS)’. Interestingly, protein isolated simultaneously from the normal adult rat liver exhibited inhibitory activity [10]. Similar hepatocyte stimulatory activity was also identified in the regenerating livers of dogs [11,12], pigs [13], and rabbits [14]. Purification attempts demonstrated that the protein with HSS activity separated on SDS-PAGE as a major (12,400 Da) and a minor (17,500 Da) band [15]. Subsequently, Francavilla and colleagues purified a similar protein with molecular mass of approximately 30 kDa from weanling rat livers, and named it ‘Augmenter of Liver Regeneration (ALR)’ [16,17]. ALR promoted liver regeneration following partial hepatectomy, ameliorated galactosamine-induced liver injury, and prevented liver pathology caused by portacaval shunt surgery in various animal models, but did not stimulate DNA synthesis in isolated hepatocytes reviewed in [18]. Subsequently, the ALR protein was sequenced, and its gene cloned in rat, mouse, and human [19,20]. The ALR gene encoded a 22 kDa protein, which exhibited activity similar to the protein extracted from hyperplastic liver in augmenting liver regeneration following partial hepatectomy in rats and in preventing portacaval shunt-induced liver atrophy in dogs [19,20]. The cloned recombinant rat ALR protein (rrALR), consistent with the earlier findings with native rat ALR [18], did not stimulate DNA synthesis in cultured rat hepatocytes [21,22].

### ALR is homologous to yeast protein ERV1

**Figure 1:**
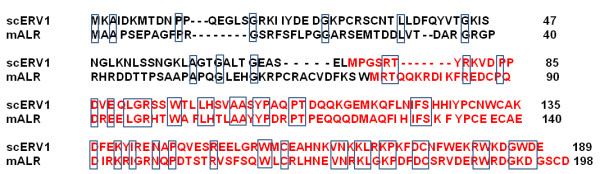
**Amino acid sequence of yeast ERV1 and mouse ALR.** Homology between the yeast and mouse proteins is shown by boxed amino acids. Red letters show amino acid sequences of the proteins identified originally [19,24] with no mitochondrial targeting sequence. These proteins, however, were found to be functionally active in augmenting liver regeneration (ALR) and maintaining cell viability (ERV1). Black letters added to the original sequences show the physiologically produced proteins that can be imported into mitochondria.

The nucleotide and amino acid sequences of the rat and mouse ALR (198 amino acids) are 96% and 90% homologous, respectively [20]. The human ALR (205 amino acids) is about 90% homologous to the rodent ALR [20,23]. The mammalian ALR exhibits significant (about 40%) homology with a protein expressed in the yeast *Saccharomyces cerevisiae* (*S. cerevisiae*) named Essential for Respiration and Viability 1 (ERV1) (Figure 1) [19,20,24]. Thus, ALR is also known as growth factor ERV1 homolog of *S. cerevisiae* (GFER). Interestingly, both ERV1 [24] and ALR [19] were initially demonstrated to be relatively small proteins with mw of approximately14 kDa and 15 kDa, respectively, and lacked typical leader sequence near the amino terminus for import into mitochondria (Figure 1, red letters). However, further investigation demonstrated that both proteins have additional amino-terminal sequences with mitochondrial import sequence and molecular size of approximately 22 kDa (Figure 1) [20,25]. The 125-amino acid and 198- or 205-amino acid forms of ALR are known as ‘short’ and ‘long’ ALR, respectively. A protein identical to human homologue of ERV1 with molecular weight of about 15 kDa was cloned from human fetal liver; this protein named ‘hepatopoietin (HPO)’ exhibits 87% homology with rat ALR [26,27], and seems to be the short form of human ALR.

Subsequent work identified another gene *ERV2* in the yeast, with 30% homology to *ERV1* at the carboxy terminal [28,29]. This protein is found in microsomes where it functions as a sulfhydryl oxidase [28]. While ERV1 has been shown to exist as a monomer as well as a dimer stabilized by the amino-terminal C30 and C33 [29,30], ERV2 lacks the cysteine residues required for dimerization. It is postulated that dimerization of ERV1 may be important for its function in physiology [29,30]. Table 1 summarizes the steps in discovery of ERV1, ERV2, and ALR genes and proteins.

**Table 1:** Both ERV1 and ALR were initially reported to be encoded by a 1.2 kb gene with protein products of 117 and 125 amino acids, respectively

**Protein**	**Species**	**Gene size**	**Chromosomal**	**Amino acids/**	**Mito. Leader**	**Function**	**Ref.**
Name			Location	Molecular weight	Sequence		
ERV1	*S. Cerevisiae*	1.2 kb	VII	117/~14 kDa	No	OxPhos/growth	24
ERV1	*S. Cerevisiae*	1.2 kb	VII	189/~22 kDa	Yes	OxPhos/growth	25
ERV2	*S. Cerevisiae*	1.2 kb	XVI	196/~22 kDa	No	Sulfhydryl oxidase	28, 29
ALR	Rat	1.2 kb	10	125/~15,081	No	HR/Anti-atrophy	19
ALR	Rat	ND	ND	198/~22 kDa	Yes	HR/Anti-atrophy	19, 20
ALR	Mouse	6.7 kb	17	198/~22 kDa	Yes	HR/Anti-atrophy	20
ALR	Human	ND	16	205/~22 kDa	Yes	HR/Anti-atrophy	20, 23

Subsequent investigation demonstrated that these proteins are larger in size with amino-terminal mitochondrial targeting sequence. Another yeast protein (ERV2 located in microsomes), homologous to ERV1, was later discovered. ALR is present as a single gene copy located on mouse chromosome 17 or human chromosome 16 in polycystic kidney disease 1 (PKD1) region [20,23]. The functions indicated for ALR and ERV proteins are the ones discovered initially. Other functions of these proteins are described in the text in relevant sections. HR, hepatic regeneration; ND, not determined; OxPhos, oxidative phosphorylation.

**Figure 2:**
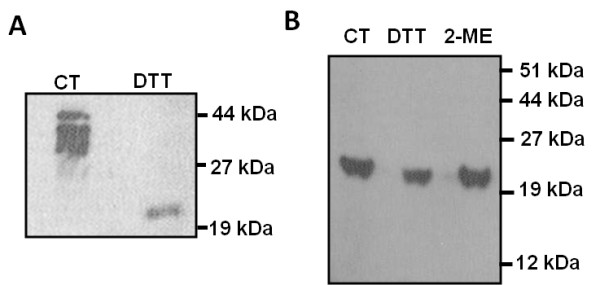
**Western blot showing native (A) and recombinant rat (rr) ALR (B). (A)** Proteins extracted from the rat liver were separated under non-reducing (control- CT) or reducing (25 mM dithiothreitol: DTT) conditions on a SDS-PAGE. The separated proteins were transferred to Immobilon-P membrane (Millipore, Bedford, MA, USA), and ALR was detected with anti-rrALR Ab and ECL kit (Amersham, UK) [21]. **(B)** The rrALR was subjected to SDS-PAGE under nonreducing (CT) or reducing (DTT or 5% 2-mercaptoethanol: 2-ME). While all of the high molecular weight ALR species (38 to 42 kDa) migrate as a single 22-kDa protein under reducing condition (A), rr-ALR migrates as a single 22-kDa protein both under reducing and non-reducing conditions.

Western blot analysis showed that a 22 kDa- and some higher molecular weight (38 to 40 kDa) proteins react with the antiserum raised against amino terminal peptide segment of the rat ALR [20]. Subsequently, western blot analysis of proteins extracted from the liver or hepatocytes under non-reducing condition demonstrated recognition of three distinct bands, with molecular weight ranging between 38 and 42 kDa, by an antibody raised against rrALR [21]. Human ALR was also found to separate into similar (38 to 42 kDa) protein species by western blot analysis [31]. Under reducing conditions, however, ALR separates as one single band with molecular weight of about 22 kDa (Figure 2A). This suggests that the native ALR is post-translationally modified, either via glycosylation or dimerization. Indeed, several O-glycosylation sites, but no N-glycosylation site, are found in the ALR molecule. Although crystallography studies using ‘short’ and ‘long’ ALR have demonstrated that the proteins can form homo-dimers [32-35], whether and how these modifications as well as post-translational alterations determine the functions of ALR in physiology and pathology remain to be determined.

### Location of ALR

ALR mRNA is expressed in heart, brain, spleen, lung, skeletal muscle, kidney, liver, and testis of rat and mouse, with maximal expression in the testes followed by the liver [19,20]. ALR is present uniformly in all regions of the liver, exclusively in hepatocytes [21]. ALR and ERV1 are present in the intermembrane space of mitochondria [25,29,36]. ALR is also located in the cytosol and nucleus [21,37,38], and ERV2 in endoplasmic reticulum [28,39]. Interestingly, unlike ERV1 that is exclusively an intracellular protein [30,40,41], ALR is secreted constitutively by hepatocytes and is present in serum [21]. Furthermore, while ALR is expressed abundantly in hepatocytes [21], the expression of ERV1 in the yeast is very low [42]. Thus the structural differences and distinct locations could be related to distinct functions of ALR, ERV1, and ERV2 proteins.

ALR is also found in all regions of the brain, co-localized with GFAP in neurons, and is specifically present in the nucleus and external envelope of mitochondria. Western blot analysis demonstrated two dimers, which under reducing condition migrated as two bands with MW of 21 and 23 kDa [42]. Low level mRNA expression of human ALR homologue was also reported in renal adenocarcinoma, cystic renal tubular epithelium, and brain [23]. Specific cellular/subcellular localization of ALR in non-hepatic organs and its functional characterization remain to be determined.

### Functions of ALR

#### *Survival and regeneration of hepatocytes*

Early experiments showed that the crude extract of regenerating or hyperplastic liver, but not the unmodified adult liver, contains ALR-like activity that augmented partial hepatectomy-induced liver regeneration and prevented portacaval shunt-induced hepatic atrophy [10,11]. Based on these observations, it was proposed that ALR is absent from the normal adult liver. However, expression of ALR mRNA and protein in various tissues, including the liver, of normal rat [19,20], and presence of equivalent amounts of ALR (mRNA and protein) in replicating and quiescent hepatocytes, *in vivo*, [21] argued for its function in physiology.

The indication that ALR might be a protein of critical physiological importance was provided by the finding that ALR’s yeast ortholog ERV1 is essential for the biogenesis of mitochondria, normal mitochondrial morphology, and stable maintenance of mitochondria in *S. cerevisiae*[24,25,40]. The mitochondrial function of ALR and ERV1 is apparent from their presence in the intermembrane space of the organelle [41]. Disruption of the *ERV1* gene or null mutation in the haploid strain led to temperature-conditional mutation resulting in alterations of the mitochondrial inner membrane, complete loss of mitochondrial genome, arrest in cell-division cycle, and death of the yeast after a few replication cycles [24,40,42]. It was suggested that high *in vivo* stability of the ERV1 mRNA and protein could be a reason for delayed death of the yeast with disrupted *ERV1* gene, despite the original low level of ERV1 expression [24].

In contrast to a lag period of a few days before the death of *S. cerevisiae* with mutated *ERV1* gene, inhibition of ALR synthesis with ALR-mRNA antisense oligonucleotide (ALR-AS) caused rapid (within hours) apoptotic/necrotic death of hepatocytes [43]. The accelerated death occurred even though significant level of residual ALR was still present in these hepatocytes. Examination of the subcellular fractions revealed strong depletion of mitochondrial ALR followed by ATP depletion in ALR-AS-transfected cells [43]. These results indicate the possibility of highly dynamic movement of ALR in and out of mitochondria. It is plausible that the arrest of ALR synthesis prevents its translocation to replace the protein that moves out of the mitochondria, thus causing its deficiency and consequent mitochondrial dysfunction. Further research is required for detailed characterization of the dynamic nature of ALR in relation to the mitochondrial and extramitochondrial localization and functions.

#### *ALR and ERV1 are functionally interchangeable*

The mitochondrial presence of ALR and ERV1, necessary for the survival of hepatocytes and *S. cerevisiae*, respectively, led to the question whether these homologous proteins are functionally interchangeable. Both the 15 kDa (short) and 22 kDa (long) cloned ALR proteins were found to prevent portacaval shunt-induced liver atrophy in dogs [19,20] suggesting that the antiatrophic/regenerative activity resides in the carboxy terminal sequence of the protein. This notion was confirmed by the finding that short form of both yeast ERV1 [24] as well as human ALR [23] that lack mitochondrial targeting amino terminal sequence rescued *S. cerevisiae* with mutated ERV1 gene from death. In addition to the absolute necessity of their carboxy terminal sequences in preventing cellular atrophy or loss of viability, these observations provided the proof that ALR and ERV1 proteins have structural and functional similarity. Indeed, growth arrest and death of *S. cerevisiae* due to deletion of carboxy terminal sequence from ERV1 could be restored by substitution with ALR’s carboxy terminal sequence [23,44]. In contrast, the amino terminal sequences, necessary for the translocation of ALR or ERV1 proteins to mitochondria, are not interchangeable. The independence from mitochondrial DNA and hence oxidative phosphorylation was proposed to be the reason why the yeast lacking mitochondrial targeting ALR/ERV1 sequence maintains viability [24,40]. However, rigorous examination of the precise roles of mitochondrial and extramitochondrial ALR/ERV1 in maintaining cell viability is required.

### Extracellular effects of ALR

#### *Effects on hepatocytes*

As mentioned above, the 22 kDa rrALR does not stimulate DNA synthesis in rat hepatocytes [21,22]. Furthermore, radioligand binding or cross-linking studies failed to demonstrate receptor for ALR on rat hepatocytes [21]. On the other hand, using radiolabeled short (15 kDa) recombinant human ALR (rhALR), Wang *et al.*[27] reported presence of high affinity ALR receptor in human and rat hepatocytes. Interestingly, on a molar basis, hepatocyte DNA synthesis stimulated by rhALR was much stronger than that by the powerful hepatocyte mitogens EGF and TGF-α [27]. The effects of rhALR and the most potent hepatocyte mitogen HGF on the DNA synthesis in human hepatocytes were also found to be comparable [45]. Why the physiological 22 kDa rat ALR protein fails to induce DNA synthesis in isolated hepatocytes, while the short human ALR is equipotent or even stronger than EGF, TGF-α, or HGF remains a topic of further investigation. One possibility is that the short rhALR may instigate intracellular signaling coupled to cell proliferation similar to these mitogens (that is, EGF, TGF-α, or HGF). In this regard, the short rhALR was reported to stimulate tyrosine-phosphorylation of the EGF receptor, and subsequent activation of mitogen-activated protein kinase in human hepatoma HepG2 cell line [46]. Since EGF receptor can bind several mitogens including some G-protein-coupled or other unidentified receptor agonists [47,48], it was proposed that the short rhALR may act in a similar manner on the EGF receptor [46]. However, an EGF receptor-independent and ERK1/2-MAPK-dependent pathway of human hepatocyte proliferation by rhALR has also been reported [49].

The short rhALR stimulates NFκB activation [50] and polyamine synthesis [45] in human hepatocytes. *In vitro*, polyamines were found to play an important role in growth factor induced DNA synthesis in cultured rat hepatocytes [51,52]. *In vivo*, polyamines are required for hepatic regeneration [53,54], and to improve the survival rate of rats after liver transplantation [55]. HGF and EGF also increase the levels of a polyamine, putrescine, during hepatocyte regeneration *in vivo* and DNA synthesis *in vitro*[56]. Thus, ALR may promote liver regeneration by elevating polyamine levels. In fact, rhALR was found to increase expression of c-Myc, and activities of ornithine decarboxylase and S-adenosylmethionine decarboxylase, which are all involved in polyamine synthesis [45]. Several groups have shown that factors such as HGF, EGF, TNF-α, IL-6, and IL-1, which activate NFκB and/or c-Myc signaling pathways, and promote liver regeneration directly or by acting as priming agents [2], inhibit cytochrome p-450 enzymes [57-67]. Upon testing whether ALR may exert similar effect, it was found that rhALR caused down-regulation of cytochrome P450 enzymes in human hepatocytes suggesting that this may be another mechanism of ALR’s role in liver regeneration [50]. Together, these findings lead to the postulate that ALR may support cell proliferation by influencing NFκB, c-Myc, polyamines, and cytochrome P-450. But the relevance of the above-described *in vitro* actions of ALR on hepatocytes to its role in their replication *in vivo* is ambiguous. Since ALR is released from the liver soon after partial hepatectomy in rats (21) and exogenously administered recombinant rat ALR augments liver regeneration in this model (22), it will be critical to investigate whether the proliferative response occurs by ALR’s direct action on hepatocytes and/or via mediators released from the non-parenchymal cells. Obviously, the attendant gene and protein expression pattern related to liver regeneration in the context of changes in hepatic and circulating ALR, and their relationship to other mitogens/co-mitogens and growth inhibitors will be the areas that should be addressed rigorously.

#### *Effects on Kupffer cells and natural killer cells*

Another important mechanism of the role of ALR in unabated liver regeneration might be its ability to prevent hepatocyte injury by affecting cells of the immune system, infiltrating inflammatory cells and resident macrophages. Earlier work demonstrated that ALR inhibits the lytic activity of hepatic natural killer (NK) cells [68]. While hepatocytes in the normal liver are resistant to the injurious effect of NK cells, those in the regenerating liver are highly sensitive [69]. Thus, it is critical that NK cells are rendered ineffective in causing injury to hepatocytes during liver regeneration. Indeed, NK cells isolated from the regenerating livers are unable to cause lysis of YAC-1 tumor cells [69]. Furthermore, serum ALR levels are found to correlate negatively with peripheral blood-derived NK cell activity in a clinical study [70]. Interestingly, the inhibitory effect of ALR on NK cells was similar to that of HGF and IGF-1 [68]. Therefore, considering the elevated levels of HGF and IGF-1 [2-5] following partial hepatectomy, the precise contribution of ALR to liver regeneration via inhibition of NK cells needs further examination.

**Figure 3:**
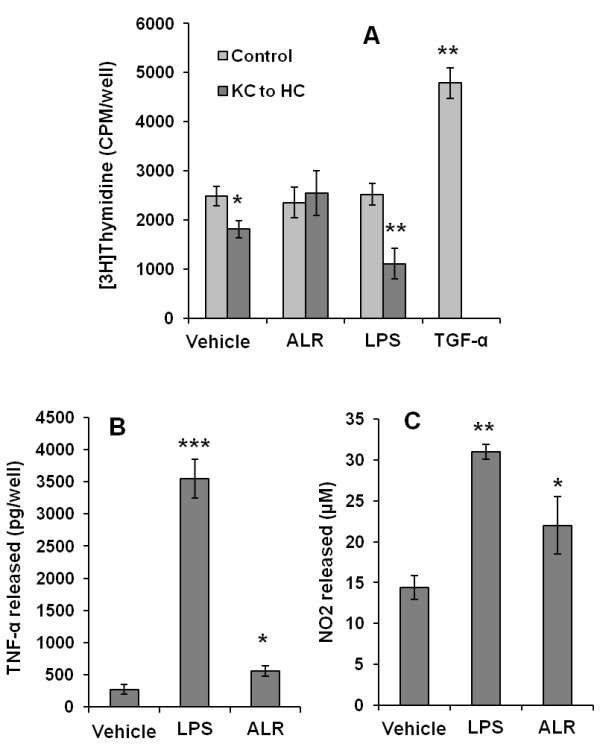
**Effect of ALR and LPS on Kupffer cells and hepatocytes. (A)** Kupffer cells (KC) were incubated with carrier (PBS), 50 nM rrALR or 100 ng/ml LPS for 24 h. The medium (KC to HC) was transferred to hepatocytes (HC). Control hepatocytes were incubated with 50 nM ALR or 100 ng/ml LPS. At 24 h, DNA synthesis (^3^H]thymidine) incorporation assay was performed. While KC-conditioned medium inhibited DNA synthesis in HC, the effect was reversed or augmented when KC were conditioned with ALR or LPS, respectively. ALR or LPS, on their own, did not affect HC DNA synthesis, while 5 nM TGF-α exerts robust effect. **(B**, **C)** Kupffer cells were incubated in the presence of 100 ng/ml LPS or 50 nM rrALR for 24 h. The medium was aspirated and TNF-α (B) or nitric oxide product NO2 (C) were measured. *P ≪ 0.05 *vs*. vehicle; **P ≪ 0.01 *vs*. vehicle or 0.05 *vs.* ALR; ***P ≪ 0.001 *vs*. vehicle or ALR (see [22] for details of the procedures).

Immediate release of ALR from the rat liver following partial hepatectomy [21] indicated that the released ALR may stimulate non-parenchymal cells to produce mediators of hepatic regeneration. This postulate was supported by the observation that Kupffer cells release TNF-α and IL-6, and NO by increasing the expression of inducible NO synthase (iNOS) upon stimulation with rrALR [22]. These effects of ALR on Kupffer cells were mediated via a cholera toxin-sensitive G-protein-coupled high affinity receptor [22]. ALR-induced synthesis of TNF-α, IL-6, and NO was much more modest compared to that stimulated by endotoxin (lipopolysaccharide (LPS)) (Figure 3). While the medium conditioned by Kupffer cells inhibited hepatocyte DNA synthesis, which was augmented when the medium was conditioned in the presence LPS (Figure 3), ALR’s presence in the medium during conditioning by Kupffer cells reversed the inhibitory effect [22] (Figure 3). These observations suggest that ALR prevents or inhibits the release of hepatocyte inhibitors by Kupffer cells, while LPS augments this process*. In vivo*, it is likely that ALR promotes liver regeneration after partial hepatectomy [18,22] by stimulating the release of priming agents (TNF-α, IL-6, and NO) while inhibiting the release of anti-mitogenic agents by Kupffer cells. It can be postulated that exogenously administered ALR provides beneficial effect reported in experimental fulminant hepatic failure [71] and cirrhosis [72] via mechanisms involving its actions on non-parenchymal cells or even on hepatocytes. This suggestion is supported by the observation that short rhALR protects human primary hepatocytes and a human hepatocyte tumor cell line (HepG2) from apoptosis induced by ethanol, TRAIL, TGF-β, and actinomycin D [73]. These effects appear to be liver specific as the short rhALR failed to elicit similar response on cell lines derived from other organs [73]. In contrast, the short rrALR was found to protect neuronal cell line SH-SY6Y from H2O2-induced death [74].

### Enzymatic activities associated with ERV1/ALR

#### *Protein sulfhydryl oxidase*

Coppock *et al*. [75] identified the presence of ALR/ERV1-homologous motif in the amino terminal portion of quiescin (QSOX) Q6 protein that belongs to a family of quiescin-sulfhydryl oxidases. Thioredoxin is another protein motif at the carboxy terminal portion of QSOX. The human QSOX (quiescin Q6) gene is located on chromosome 1q25.2, and has 12 exons comprising more than 41,000 bp, and is highly transcribed in fibroblasts during their quiescence [75]). Several proteins and peptides, but not monothiols such as glutathione, are substrates for QSOX; QSOX has a widespread presence in endoplasmic reticulum and golgi of eukaryotes, and it is also secreted by the cells [76-79]. However, *S. cerevisiae* does not express QSOX. These observations suggested that ALR/ERV1 may also function as sulfhydryl oxidase. Indeed, ERV1 and ERV2 [29,30,39,40,80] as well as purified ALR [81] were found to contain FAD-linked sulfhydryl oxidase activity. Crystal structures of these proteins revealed a helix-rich fold in the carboxy terminus containing the core sulfhydryl oxidase activity with a FAD-binding site and a conserved proximal CxxC motif [33,40,82]. Thus both short and long ALR are able to perform this function. Mutation of either of two cysteine residues, that are located adjacent to the FAD, results in inactivation of ALR’s sulfhydryl oxidase activity [33]. However, ALR and ERV1 are relatively weak sulfhydryl oxidases on their own [29,30,40,81,82]. Perhaps ALR and ERV1 function as specialist sulfhydryl oxidase for specific cellular substrates. It has been proposed that ALR might introduce disulfides found in proteins located in the mitochondrial intermembrane space, including thionein [83-85], TIM10 [85], TIM13 [86], and superoxide dismutase [87]. A functional aspect of ALR-associated sulfhydryl oxidase activity was recently reported in hepatic organogenesis in zebra fish. Using morpholino antisense oligonucleotide technique, Li *et al.*[88] showed that ALR-antisense suppressed liver outgrowth in zebra fish, and this could be rescued by ALR overexpression, but only partly when mutant ALR lacking sulfhydryl oxidase activity was used.

#### *Cytochrome c reductase*

Allen *et al.*[89] reported an important role of ERV1 in the biogenesis of small proteins imported via the Mia40-dependent pathway in the mitochondrial intermembrane space. ERV1 [89] and ALR [34] can also act as reductant for cytochrome c that is imported via Mia40-independent pathway [90,91]. Functional link of the Mia40-dependent pathway to the distinct import pathway of cytochrome c may thus create a route for the transfer of electrons from the incoming precursor to cytochrome c as an acceptor. The abundance of cytochrome c in mitochondrial intermembrane space argues for its function as potential oxidant for ALR *in vivo*[34]. Such oxidation of ALR coupled to the respiratory chain can provide a mechanism for prevention or reduced generation of H2O2. Based on these observations, it is postulated that interactions between ALR/ERV1 (oxidation) and cytochrome c (reduction) could be critical in regulating cell viability as shown by inability of the yeast cells with mutated cytochrome c to grow under anaerobic conditions [89].

#### *Fe/S maturation of proteins*

Another function associated with the ALR molecule is its ability to induce Fe/S maturation of cellular proteins. The entire ERV1/ALR sequence, including the poorly conserved amino terminus, is required for ALR/ERV1 to catalyze Fe/S assembly of cytosolic but not mitochondrial proteins [41]. Replacement of the amino terminus (1 to 81) of mammalian ALR (205 aa) with 1 to 93 amino acid amino terminus sequence of yeast ERV1 enables the fusion protein to translocate into yeast mitochondria and function as Fe/S maturation enzyme [41]. This effect of ALR may not just be limited to hepatocytes. In fact, ALR has also been shown to accumulate in mitochondria of spermatogonia and spermatocytes, where it is suggested to play a role in assembly of Fe/S in mitochondrial membrane proteins leading to complex mitochondrial changes occurring during spermatogenesis [36]. However, it should be noted that the enzymatic activity of ERV1 to catalyze Fe/S assembly of cytosolic protein is relatively weak [30,37]. Additional research should shed light on whether ALR can also cause Fe/S maturation of mitochondrial proteins, particularly the proteins of respiratory chain complexes, given its putative role in oxidative phosphorylation.

#### *ALR and pathology*

Considering the abundance of ALR in the liver and its secretory nature, researchers have examined changes in circulating and tissue levels of ALR in certain pathological conditions. Tanigawa *et al*. [70] found significantly increased serum ALR levels in patients with acute and fulminant hepatitis; ALR was modestly increased in chronic hepatitis and liver cirrhosis. These data and immediate release of ALR in circulation following partial hepatectomy [21] suggest that alterations in serum ALR levels could provide an indication of hepatocyte stress or injury. In this regard, we have found that LPS increases ALR release from cultured hepatocytes and circulating ALR levels increase in rats during endotoxemia (unpublished work).

Hepatic ALR increases rapidly but transiently following portacaval shunt in rats, accompanied by increases in HGF, TGF-α, and TGF-β [92]. Despite the increases in protein levels of these growth factors (ALR, HGF, and TGF-α), the liver undergoes atrophy. However, exogenously administered ALR, HGF, or TGF-α prevent the pathological changes of portacaval shunt [18]. These results indicate that the levels endogenously produced pro-mitogenic or anti-apoptotic factors are not sufficient to ameliorate pathological development or their actions are suppressed by concomitantly produced anti-mitogenic/pro-apoptotic factors such as TGF-β.

Increased mRNA and protein expression of ALR was observed in liver from patients with cirrhosis, and hepatocellular and cholangiocellular carcinoma [31]. Predominant expression of ALR was found in hepatocytes and cholangiocytes of normal, cirrhotic, or cancerous liver tissue [31]. It will be of interest to find out whether elevated ALR in cancerous or regenerating cirrhotic hepatocytes is a causal or consequence of the disease. Whether excessive expression of ALR in hepatocytes, that already contain this protein in abundance, drives them to become neoplastic may be addressed by generating mice over-expressing liver-specific ALR.

An area of investigation that needs to be explored is the pathological developments in the liver due to mitochondrial ALR deficiency. Given its role in oxidative phosphorylation, mitochondrial ALR deficiency might induce oxidative stress causing abnormal hepatocyte functions. In this regard, we have found that mitochondrial ALR deficiency causes hepatocyte injury [43] and accumulation of lipids (unpublished work). Although ALR deficiency-induced oxidative stress may be a primary mechanism of lipid accumulation, the possibility that mitochondrial ALR may directly affect fatty acid metabolism should be considered. ALR deficiency causes apoptosis/necrosis of hepatocytes, which induces inflammatory response. Inflammatory mediators are known to induce activation of stellate cells and consequent fibrogenic activity [93,94]. It is also reported that engulfment of apoptotic hepatocytes by stellate cells can induce their activation [95]. Thus, it is tempting to speculate that genetic deficiency of ALR may place human subjects at high risk to develop steatohepatitis (alcoholic and non-alcoholic), a topic of high clinical relevance.

## Conclusions

ALR is an enigmatic protein whose functional characteristics and their underlying mechanisms are not well understood as yet despite the fact that the protein was discovered more than 35 years ago and its gene cloned 18 years ago. Originally identified as an augmenter of liver regeneration, and assumed to be present only in the hyperplastic liver, we now know that ALR is present ubiquitously in multiple forms among eukaryotes, and its presence in various subcellular organelles and extracellular compartments suggests that it may have distinct and important physiological functions. Although physiologically present as a 22 kDa protein, a large body of work has been done using the short form of ALR (15 kDa) or its dimer. Curiously, the short ALR has been shown to be equipotent in stimulating DNA synthesis in hepatocytes as HGF and TGF-α, while the 22 kDa protein is unable to stimulate this reaction. This critical observation begs a question whether the mitogenic property of short human ALR could be exploited as a therapy necessitating liver regeneration in pathological conditions. Certainly, ALR plays a critical role in maintaining viability of hepatocytes, and whether it performs similar function in cells of other organs remains to be determined. Although enzymatic activities (sulfhydryl oxidase, Fe/S assembly, and cytochrome c reductase) are associated with ALR, they are relatively weak as compared to that of other known enzymes. It is possible that ALR is highly selective in performing its enzymatic function for specific proteins. Clearly, significant further work has to be done to understand the multiple roles of this protein. Perhaps genetically engineered mouse models (lacking or over-expressing ALR in specific organs) will be required to reveal its functions.

## Abbreviations

ALR: Augmenter of liver regeneration; EGF: Epidermal growth factor; ERV: Essential for respiration and viability; GFER: Growth factor ERV-like; HGF: Hepatocyte growth factor; IGF: Insulin-like growth factor; IL: Interleukin; NO: Nitric oxide; rrALR: Recombinant rat ALR; rhALR: Recombinant human ALR.

## Competing interests

The author does not have any financial competing interests to disclose. The content of this article does not represent the views of the Department of Veterans Affairs or the United States Government.
